# Hospitalizations for staphylococcal scalded skin syndrome in Spain (2016–2023): unequal disease burden concentrated in infants

**DOI:** 10.3389/fped.2026.1888820

**Published:** 2026-08-26

**Authors:** Isabel Belinchón-Romero, José-Manuel Ramos-Rincón

**Affiliations:** 1 Department of Clinical Medicine, Miguel Hernández University, Alicante, Spain; 2 Department of Dermatology, Dr. Balmis General University Hospital, Alicante, Spain; 3 Alicante Institute for Health and Biomedical Research (ISABIAL), Alicante, Spain; 4 Division of Infectious Disease, Dr. Balmis General University Hospital, Alicante, Spain; 5 Department of Internal Medicine, Dr. Balmis General University Hospital, Alicante, Spain

**Keywords:** adults, children, hospitalization, skin infections, Spain, staphylococcal scalded skin syndrome

## Abstract

**Aim:**

This study aimed to describe the epidemiological characteristics and direct healthcare costs of hospitalizations resulting from staphylococcal scalded skin syndrome (SSSS) in Spain, stratified by age group.

**Materials and methods:**

This retrospective, population-based study included all patients hospitalized with SSSS in Spain between 2016 and 2023. Data were obtained from the Spanish National Hospital Discharge Database and stratified into three age groups for the main analysis: 0–4 years, 5–14 years, and ≥15 years. Outcomes were hospitalization rates per 100,000 admissions and per 1,000,000 population. Temporal trends were assessed using linear regression.

**Results:**

Children aged 0–4 years accounted for most the 1100 SSSS-related hospitalizations (*n* = 817, 74.3%), followed by those aged 5–14 years (*n* = 199, 18.1%) and adults (*n* = 84, 7.6%). Hospitalizations increased 278% over the study period (*n* = 73 in 2016 vs. *n* = 276 in 2023, p for trend <0.001). Hospitalization rates followed a similar pattern (p for trend <0.001), both per 100,000 admissions (from 1.66 to 5.92) and per 1,000,000 pop. (from 1.57 to 5.71). The highest rates were consistently observed in children aged 0–4 years, reaching 119.04 per 1,000,000 pop. in 2023. Forty-nine children (4.9%) were admitted to the intensive care unit (ICU), with significantly higher rates in newborns (19.6%; *p* < 0.001). Two newborns (4.3%) and 16 (19.0%) adults died during admission (1.6%). Adults had more comorbidities and experienced worse clinical outcomes compared to children.

**Conclusions:**

SSSS hospitalizations in Spain are rising, primarily affecting young children. Severity varies by age and is most pronounced in infants and adults.

## 1 Introduction

Staphylococcal scalded skin syndrome (SSSS) is an acute exfoliative dermatosis caused by the systemic dissemination of exfoliative toxins produced by *Staphylococcus aureus* (1–3). These toxins target desmoglein-1, leading to intraepidermal cleavage, which presents clinically as diffuse erythema, flaccid blistering, and subsequent desquamation, typically associated with a positive Nikolsky sign (1, 3). SSSS is a toxin-mediated disease in which exfoliative toxins act as serine proteases, disrupting keratinocyte adhesion in the superficial epidermis (3).

SSSS predominantly affects newborns and young children, particularly those under 5–6 years of age, due to their immature immune system and reduced renal clearance of circulating toxins (1–4). Host-related factors such as limited antibody response to exfoliative toxins and specific susceptibility mechanisms also contribute to disease development in young children (1–4).

SSSS is primarily a pediatric disease, but rarely, it can also occur in adults (5, 6), usually associated with underlying conditions like immunosuppression, chronic comorbidities, or kidney failure; in this population, it is linked to worse clinical outcomes than in children (5, 6). The clinical spectrum of SSSS ranges from localized skin involvement to a generalized form with extensive epidermal detachment (1–6).

Despite its clinical relevance, epidemiological data on SSSS remain limited, particularly at the population level (1, 2). Most available studies are based on case series or single-center experiences (5, 7, 8), whereas large-scale analyses evaluating hospitalization trends, clinical characteristics, and healthcare burden are scarce (1, 2). Furthermore, there is a lack of standardized, evidence-based recommendations in specific populations such as newborns, reflecting gaps in the current literature (2). This is especially true in Spain, where nationwide data on SSSS hospitalizations in both pediatric and adult populations are lacking.

Given that childhood represents a unique epidemiological setting with specific susceptibility factors and healthcare implications, it is important to better characterize the burden of SSSS in this group (3). At the same time, describing adult cases, although infrequent (5), may provide valuable insights into differences in clinical presentation and outcomes across age groups.

Therefore, the aim of this study was to estimate hospitalization rates, describe the clinical characteristics, and assess the economic impact of SSSS in the pediatric and adult population in Spain, while also providing a descriptive analysis of hospitalized adult cases using a nationwide hospital discharge database.

## 2 Methods

### Study design

This population-based, cross-sectional study included pediatric and adult patients who were hospitalized with SSSS in any public and private hospital in Spain from 1 January 2016 to 31 December 2023. Data were obtained from the Spanish Registry of Specialist Care Activities, maintained by the Spanish Ministry of Health. This registry includes the minimum basic dataset for hospital discharges, which contains demographic, administrative, and clinical data, including diagnoses and procedures performed for all hospitalizations in Spain. All diagnoses and procedures were coded according to the International Classification of Diseases, 10th Revision (ICD-10) (9). The Ministry of Health periodically audits the registry to ensure data quality and accuracy.

Data collected included discharge dates and information on SSSS hospitalizations, coded using the ICD-10 code: L00 (staphylococcal scalded skin syndrome). Cases were identified exclusively through this ICD-10 discharge diagnosis code recorded in the Spanish Registry of Specialist Care Activities database. Although, to our knowledge, no specific validation studies assessing the accuracy of SSSS coding within this registry have been published, the database undergoes regular quality control and auditing procedures conducted by the Spanish Ministry of Health to promote the completeness and consistency of coded hospital discharge data.

### 2.2 Variables

Explanatory variables comprised (a) demographic variables: age group (0–4, 5–14 years, ≥15 years) and sex; (b) clinical characteristics: type and location of SSSS (by ICD-10 code), and comorbidities (e.g., lymphoma, malignancies, obesity, diabetes, HIV, and primary immunodeficiencies; (c) clinical complications: sepsis, severe sepsis, and acute kidney injury; (d) outcomes: intensive care unit (ICU) admission, length of stay, in-hospital mortality, and (e) cost of hospitalization. Hospitalization costs were estimated using the diagnosis-related group (DRG) system. Hospitalization costs were estimated using the Diagnosis-Related Group (DRG) system, as employed in the Spanish National Hospital Discharge Database (10). DRGs classify hospital admissions into clinically similar groups expected to consume comparable healthcare resources, taking into account factors such as the primary diagnosis, procedures performed, comorbidities, complications, age, and length of stay. For each hospitalization, an estimated cost was assigned based on the corresponding DRG. Thus, the costs analyzed in this study correspond to patient-level estimated costs recorded in the database rather than standardized costs derived solely from length of stay or ICU admission.

Table 1 includes the full list of ICD-10 codes for diagnoses and comorbidities. Sepsis was identified using ICD-10 codes A40.x (streptococcal sepsis) and A41.x (other bacterial sepsis, including *S. aureus*, *Haemophilus influenzae*, anaerobes, and other Gram-negative organisms). These codes were used to identify the occurrence of sepsis during hospitalization, regardless of the causative pathogen, rather than to attribute sepsis specifically to *Staphylococcus aureus* infection. Severe sepsis was defined using R65.2, indicating infection with acute organ dysfunction. The underlying infection was always coded first.

**Table 1:** International classification of diseases, 10th edition codes used in the study.

Diagnosis	Code
Staphylococcal scalded skin syndrome (SSSS)	L00
Comorbidities	
Diabetes mellitus	E10.x; E11.x; E13.x
Neoplasms (solid tumors)	C00.x–C80.x
Leukemia	C92–C96
Lymphoma	C81–C91
Obesity	E66.x; Z68.3; Z68.4
Hypertension	I10–I15
Dyslipidemia	E78.x
Chronic kidney disease	N18.x
Heart failure	I50.x
Ischemic heart disease	I20–I25
Cirrhosis	K74.x
Alcohol use	F10.x
Smoking	F17.x; Z72.0
Transplantation	Z94.x
Immunodeficiencies	B80.x; B81.x; B82.x; B83.x; B84.x; D70.x
Concomitant conditions affecting the dermis	
Trauma and open wounds	S00.x–S99.x
Other injuries/unspecified trauma	T14.x
Atopic dermatitis	L20.x
Psoriasis	L40.x
Complications	
Sepsis	A40.x; A41.x
Severe sepsis	R65.2x
Acute kidney injury	N17.x

In this dataset, a coded diagnosis of microbial infection was interpreted as culture-confirmed in the clinical setting (blood or another sterile site). However, the database does not provide access to individual microbiological reports, precluding the verification of culture source and method of isolation (Table 1).

### 2.3 Statistical analysis

The hospitalization rate per 100,000 admissions (HR_ad_) was calculated as the number of SSSS hospitalizations per 100,000 all-cause hospital admissions per year, using data from the hospital discharge database (10). In addition, the hospitalization rate per 1,000,000 population (HR_pop_) was calculated as the number of SSSS hospitalizations per 1,000,000 population using age-adjusted annual population estimates from the Spanish National Institute of Statistics (11). Temporal trends in SSSS hospitalizations were analyzed using linear regression models, with calendar year included as a continuous independent variable. Separate models were fitted for absolute counts of hospitalizations and for HR_ad_ and HR_pop_. The statistical significance of temporal trends was assessed using the *p*-value of the regression coefficient (p for trend). Relative changes over time were quantified as percentage change between the first (2016) and last (2023) years of the study period. Analyses were conducted overall and stratified by age group (0–4 years, 5–14 years, and ≥15 years). Subsequently, children in the youngest age group were further stratified by smaller age bands (0–28 days, 29–365 days, 1 year, 2 years, 3 years, 4 years, and ≥5 years old).

Categorical variables were reported as absolute counts and percentages. Continuous variables were summarized as medians with interquartile ranges (IQRs). Normality was assessed using the Kolmogorov–Smirnov test. Comparisons across groups were performed using the chi-squared test for categorical variables and the Mann Whitney U or Kruskal–Wallis tests for continuous variables. *P* values under 0.05 were considered statistically significant. Analyses were conducted using IBM SPSS for Windows, version 25.0 (IBM Corp., Armonk, NY, USA).

### 2.4 Ethical aspects

The Spanish Ministry of Health provided the anonymized dataset after removing all personal identifiers. According to Spanish legislation, informed consent is not required for studies using anonymized administrative databases. The Ethics and Research Integrity Committee of Miguel Hernández University approved the research protocol (Ref. AUT.DMC.JMRR.241103), which adhered to the ethical principles of the revised Declaration of Helsinki (2013).

## 3 Results

### 3.1 Annual trends in case numbers and hospitalization rates by age group

From 2016 to 2023, there were a total of 35.55 million hospital admissions (1.59 million aged 0–4 years, 0.93 million aged 5–14 and 32.98 aged 15–105 years). Of these, 1100 admissions were coded as SSSS: 817 (74.2%) were in 0–4-year-olds, 199 (18.1%) in 5–14-year-olds, and 84 (7.6%) adults aged 15 or older.

Temporal data show a clear ascending trend in hospitalizations over time (Table 2). The annual number of hospitalizations rose from 73 cases in 2016 to 276 in 2023, representing an overall increase of 278% (*p* = 0.005). This increase was more pronounced in children aged 5–14 years (+436.4%, *p* = 0.014), followed by children aged 0–4 years (+346.8%, *p* = 0.003). In contrast, the number of hospitalizations decreased among adults (−53.3%; *p* = 0.103).

**Table 2:** Episodes of staphylococcal scalded skin syndrome (SSSS) hospitalization in pediatric and adult populations, hospitalization rates per 100,000 all-cause admissions (HR_ad_) and per 1,000,000 inhabitants (HR_pop_) by age group in Spain.

Year	Total			0–4 years			5–14 years			≥15 years		
	*N*	HR_ad_	HR_pop_	*N*	HR_ad_	HR_pop_	*N*	HR_ad_	HR_pop_	*N*	HR_ad_	HR_pop_
2016	73	1.66	1.57	47	20.58	21.77	11	8.90		15	0.37	0.38
2017	90	1.97	1.94	67	29.84	31.77	15	11.93	2.28	8	0.19	0.20
2018	105	2.32	2.25	77	35.47	37.26	15	12.24	3.10	13	0.31	0.33
2019	133	2.92	2.82	92	43.46	45.56	28	22.88	3.10	13	0.31	0.32
2020	104	2.58	2.20	81	53.15	41.43	15	15.94	5.80	8	0.21	0.20
2021	132	3.06	2.79	99	58.12	53.20	22	21.60	3.11	11	0.27	0.27
2022	187	4.21	3.91	144	73.83	79.59	34	31.20	4.61	9	0.22	0.22
2023	276	5.92	5.71	210	109.70	119.04	59	46.21	7.14	7	0.16	0.17
Mean	118	3.08	2.90	85	53.02	53.70	19	21.36	12.51	10	0.26	0.26
% change	+278%	+257%	+264%	+347%	+433%	+446%	+436%	+419%	+449%	−53%	−59%	−55%
p-trend	**0.005**	**0**.**002**	**0**.**005**	**0.003**	**<0**.**001**	**0**.**002**	**0.014**	**0**.**002**	**0**.**014**	0.103	0.097	0.084

HR_ad_: SSSS hospitalizations per 100,000 all-cause admissions per year.

HR_pop_: SSSS hospitalizations per 1,000,000 population per year.

Regarding hospitalization rates (Table 2), over the study period, mean HR_ad_ was 3.08 and HR_pop_, 2.90. However, both rates showed a consistent upward trend, confirmed by temporal trend analysis. HR_ad_ increased by 257% (*p* = 0.002) and HR_pop_ by 264% (*p* = 0.005). This increase was driven by the rising burden in the pediatric population.

**Figure 1:**
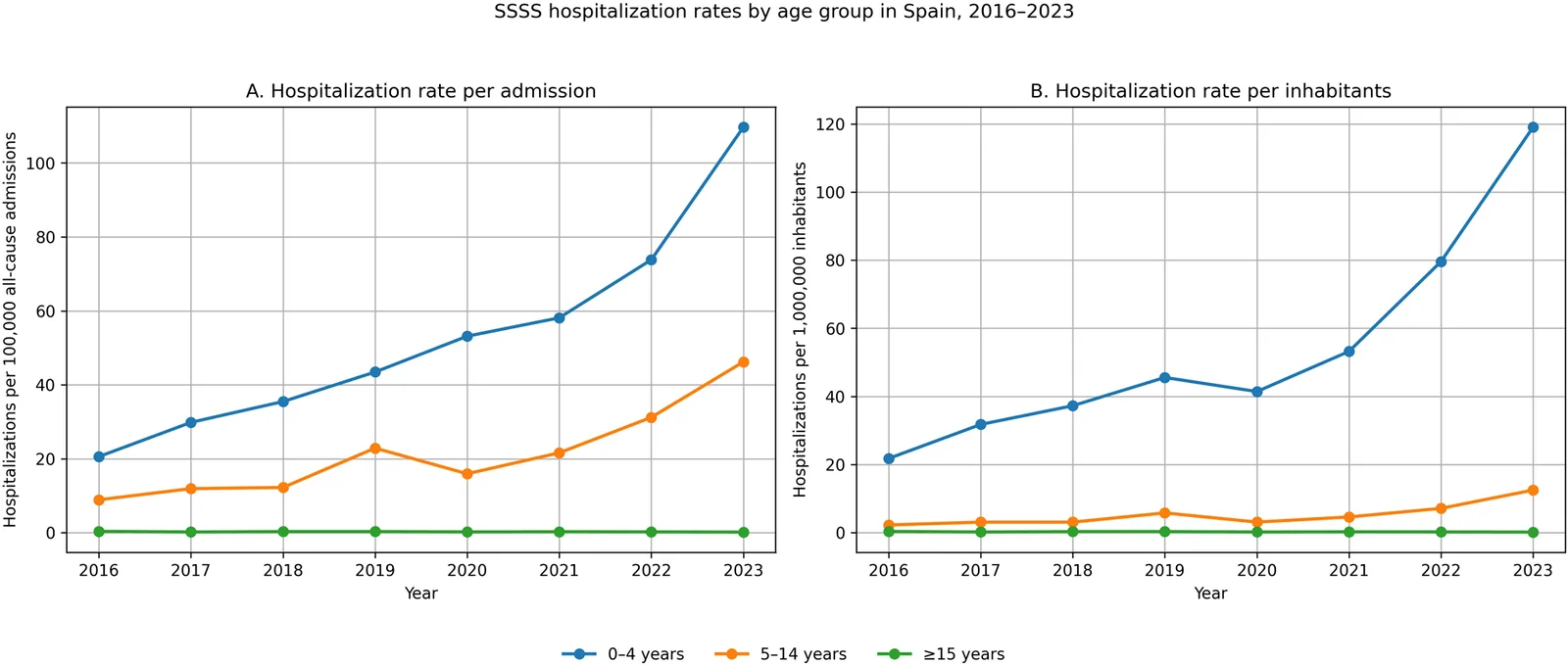
Annual hospitalization rates for staphylococcal scalded skin syndrome (SSSS) in Spain from 2016 to 2023, stratified by age group. (**A**) Hospitalization rates per 100,000 all-cause hospital admissions. (**B**) Hospitalization rates per 1,000,000 inhabitants.

When stratified by age, hospitalization rates were markedly higher in younger children. For HR_ad_, the mean rate was 53.02 in children aged 0–4 years, compared to 21.36 in those aged 5–14 years and 0.26 in adults. A similar pattern was observed for HR_pop_, with mean rates of 53.70, 5.21, and 0.26, respectively (Figure 1). In children aged 0–4 years, HR_ad_ increased by 433% (*p* < 0.001) and HR_pop_ by 447% (*p* = 0.002). Similarly, in children aged 5–14 years, HR_ad_ increased by 419% (*p* = 0.002) and HR_pop_ by 449% (*p* = 0.014). In contrast, rates in adults decreased over the study period, with no statistically significant trends observed.

### 3.2 Clinical characteristics, severity and outcomes by age group

The pediatric population showed a low prevalence of comorbidities. Among those observed, atopic dermatitis and a history of trauma were the most frequent. Clinical outcomes in children were generally favorable, with very low mortality rates, ICU admission rates below 5%, and a median length of hospital stay of approximately 4 days.

In contrast, the adult population presented a substantially and significantly higher burden of comorbidities, including hypertension, dyslipidemia, chronic kidney disease, solid and hematological malignancies, diabetes mellitus, and history of transplantation (Table 3). Clinical complications were also markedly more frequent in adults, with sepsis occurring in 19.0% of cases and acute kidney injury in 23.8%. This was associated with significantly worse outcomes, including a mortality rate of 19.0%, ICU admission in 22.0% of patients, and a longer median hospital stay, of 10 days (Table 3).

**Table 3:** Clinical, epidemiological characteristics and outcomes of staphylococcal scalded skin syndrome (SSSS) by age group in Spain.

Variable	Total *N* = 1,100	(A) 0–4 years *N* = 817	(B) 5–14 years *N* = 199	(C) ≥ 15 years *N* = 84	p:A vs. B	p:A vs. C	p:B vs. C
Male sex, n (%)	558 (50.7)	410 (50.2)	106 (53.3)	42 (50.0)	0.44	0.96	0.63
Comorbidities, n (%)							
Atopic dermatitis	73 (6.6)	55 (6.7)	15 (7.5)	3 (3.6)	0.66	0.24	0.19
Hypertension	29 (2.6)	0 (0.0)	2 (1.0)	27 (32.1)	0.03	**<0**.**001**	**<0**.**001**
Dyslipidemia	15 (1.4)	0 (0.0)	0 (0.0)	15 (17.9)	1.00	**<0**.**001**	**<0**.**001**
Chronic kidney disease	12 (1.1)	1 (0.1)	1 (0.5)	10 (11.9)	0.28	**<0**.**001**	**<0**.**001**
Diabetes mellitus	11 (1.0)	1 (0.1)	2 (1.0)	8 (9.5)	0.09	**<0**.**001**	**<0**.**001**
Trauma	9 (0.8)	4 (0.5)	1 (0.5)	4 (4.8)	1.00	**0**.**004**	**0**.**028**
Transplant	8 (0.7)	1 (0.1)	1 (0.5)	6 (7.1)	0.35	**<0**.**001**	**0**.**003**
Heart failure	8 (0.7)	1 (0.1)	0 (0.0)	7 (8.3)	0.63	**<0**.**001**	**<0**.**001**
Obesity	6 (0.5)	1 (0.1)	0 (0.0)	5 (6.0)	0.64	**<0**.**001**	**0**.**01**
Solid neoplasm	6 (0.5)	1 (0.1)	0 (0.0)	5 (6.0)	1.00	**<0**.**001**	**0**.**002**
Alcohol use	5 (0.5)	0 (0.0)	0 (0.0)	5 (6.0)	1.00	**<0**.**001**	**<0**.**001**
Smoking	5 (0.5)	0 (0.0)	1 (0.5)	4 (4.8)	0.19	**<0**.**001**	**0**.**02**
Psoriasis	4 (0.4)	1 (0.1)	0 (0.0)	3 (3.6)	0.61	**0**.**003**	**0**.**03**
Leukemia/lymphoma	5 (0.5)	0 (0.0)	1 (0.5)	4 (4.8)	0.20	**<0**.**001**	0.080
Cirrhosis	3 (0.3)	0 (0.0)	0 (0.0)	3 (3.6)	1.00	**<0**.**001**	**0**.**025**
Immunodeficiency	2 (0.1)	1 (0.1)	1 (0.5)	0 (0.0)	1.00	1.00	1.00
Ischemic heart disease	2 (0.2)	0 (0.0)	0 (0.0)	2 (2.4)	1.00	0.01	0.08
Clinical complications, n (%)							
Sepsis	20 (1.8)	1 (0.1)	3 (1.5)	16 (19.0)	0.025	**<0**.**001**	**<0**.**001**
Severe sepsis	15 (1.4)	0 (0.0)	1 (0.5)	14 (16.7)	0.20	**<0**.**001**	**<0**.**001**
Acute kidney injury	20 (1.8)	0 (0.0)	0 (0.0)	20 (23.8)	1.00	**<0**.**001**	**<0**.**001**
Outcomes, n (%)							
Mortality	18 (1.6)	2 (0.2)	0 (0.0)	16 (19.0)	0.53	**<0**.**001**	**<0**.**001**
ICU admission	67 (6.2)	42 (5.3)	7 (3.6)	18 (22.0)	0.36	**<0**.**001**	**<0**.**001**
Length of stay, days, median (IQR)	4 (3–6)	4 (3–6)	4 (3–6)	10 (6–17.5)	0.36	**<0**.**001**	**<0**.**001**
Direct cost, €							
Absolute cost	4,424,729	2,950,659	714,620	759,450	-	**-**	**-**
Median cost per admission (IQR)	3,036 (2,894–3,435)	2,913 (2,894–3,435)	2,962 (2,860–3,435)	4,086.5 (3,245.5–7,499.0)	0.32	**<0**.**001**	**<0**.**001**

ICU, intensive care unit; IQR, interquartile range. In bold, *p* value < 0.001.

### 3.3 Clinical characteristics, severity and outcomes in pediatric patients by age group

When pediatric patients were further stratified by age group (Table 4), no significant differences were observed in the distribution of comorbidities, all of which were infrequent. In contrast, relevant differences emerged in terms of severity and clinical outcomes. Mortality was exclusively observed in infants under 1 year, with the highest proportion in newborns (4.3% of those aged 0–28 days). Similarly, ICU admissions were markedly more frequent in younger patients, particularly in newborns (19.6%) and infants aged 29–365 days (7.6%), compared to older children, in whom ICU admission rates remained below 5%.

**Table 4:** Clinical characteristics, severity, and outcomes of pediatric patients with *staphylococcal* scalded skin syndrome (SSSS) by age group in Spain.

Variable	Total	0–28 days (*n* = 47)	29–365 days (*n* = 134)	1-year-olds (*n* = 147)	2-year-olds (*n* = 124)	3-year-olds (*n* = 186)	4-year-olds (*n* = 179)	5–14 years (*n* = 197)	p
Comorbidities, n (%)									
Atopic dermatitis	70 (6.9)	0 (0.0)	7 (5.2)	9 (6.1)	11 (8.9)	15 (8.1)	13 (7.3)	15 (7.6)	0.48
Diabetes mellitus	3 (0.3)	0 (0.0)	0 (0.0)	0 (0.0)	0 (0.0)	0 (0.0)	1 (0.6)	2 (1.0)	0.45
Hypertension	2 (0.2)	0 (0.0)	0 (0.0)	0 (0.0)	0 (0.0)	0 (0.0)	0 (0.0)	2 (1.0)	0.22
Chronic kidney disease	2 (0.2)	0 (0.0)	0 (0.0)	0 (0.0)	0 (0.0)	1 (0.5)	0 (0.0)	1 (0.5)	0.77
Immunodeficiency	2 (0.2)	0 (0.0)	0 (0.0)	0 (0.0)	1 (0.8)	0 (0.0)	0 (0.0)	1 (0.5)	0.59
Neoplasia	1 (0.1)	0 (0.0)	0 (0.0)	1 (0.7)	0 (0.0)	0 (0.0)	0 (0.0)	0 (0.0)	0.43
Psoriasis	1 (0.1)	0 (0.0)	0 (0.0)	0 (0.0)	0 (0.0)	1 (0.5)	0 (0.0)	0 (0.0)	0.62
Heart failure	1 (0.1)	0 (0.0)	0 (0.0)	0 (0.0)	0 (0.0)	1 (0.5)	0 (0.0)	0 (0.0)	0.62
Clinical complications, n (%)									
Sepsis	4 (0.4)	0 (0.0)	0 (0.0)	1 (0.7)	0 (0.0)	0 (0.0)	0 (0.0)	3 (1.5)	0.16
Severe sepsis	1 (0.1)	0 (0.0)	0 (0.0)	0 (0.0)	0 (0.0)	0 (0.0)	0 (0.0)	1 (0.5)	0.66
Outcomes, n (%)									
ICU admission	49 (4.9)	9 (19.6)	10 (7.6)	7 (5.0)	3 (2.5)	7 (3.8)	6 (3.4)	7 (3.6)	**<0**.**001**
Mortality	2 (0.2)	2 (4.3)	0 (0.0)	0 (0.0)	0 (0.0)	0 (0.0)	0 (0.0)	0 (0.0)	**<0**.**001**
Length of stay, median (IQR)	4 (3–6)	7 (5–10)	4.5 (4–6)	4 (3–6)	4 (3–5)	4 (3–5)	4 (3–5)	4 (3–5)	**<0**.**001**
Direct cost of admissions (€)									
Absolute cost	3,659,532	438,347	438,270	493,250	400,684	603,276	576,832	708,873	—
Median cost per admission (IQR)	2,913 (2,894–3,435)	3,435 (2,894–5,814.5)	2,913 (2,860–3,435)	2,978 (2,860–3,435)	2,913 (2,860–3,435)	2,913 (2,894–3,435)	2,913 (2,904–3,435)	2,962 (2,860–3,435)	0.15

ICU, intensive care unit; IQR, interquartile range. In bold *p* value < 0.001.

Length of hospital stay also varied significantly by age group (*p* < 0.001) and was longer in younger children. Newborns had the longest median hospital stay (7 days, IQR 5–10), followed by infants aged 29–365 days (4.5 days, IQR 4–6), whereas children older than 1 year showed a shorter and less variable length of hospital stay (median 4 days, IQR 3–6).

### 3.4 Direct healthcare costs of SSSS hospitalizations by age group

The total direct cost of the 1,100 SSSS-related hospitalizations over the study period was estimated at €4.42 million, distributed as follows by age group: €2.95 million in the 0–4 year group, €0.71 million in the 5–14-year group, and €0.76 million in adults (≥15 years) (Table 3). The median cost per admission was €3,036, with relatively similar costs observed between pediatric groups (€2,913 in 0–4 years and €2,962 in 5–14 years) and higher costs in adults (€4,087).

Within the pediatric population, the total direct cost amounted to €3.67 million, with the median cost per admission standing at €2,913; *p* = 0.15), Notably higher median costs and greater variability were observed in newborns (0–28 days: €3,435, Table 4).

## 4 Discussion

This nationwide study provides three key findings: (i) SSSS hospitalizations steadily increased over time, albeit with a temporary interruption during the COVID-19 pandemic; (ii) children under 5 years of age consistently accounted for the highest burden of hospitalization; and (iii) adults experienced substantially worse clinical outcomes despite representing only a small proportion of cases.

This nationwide study provides three key findings: (i) SSSS hospitalizations steadily increased over time, albeit with a temporary interruption during the COVID-19 pandemic; (ii) children under 5 years of age consistently accounted for the highest burden of hospitalization; and (iii) adults experienced substantially worse clinical outcomes despite representing only a small proportion of cases.

The temporal analysis demonstrated a progressive increase in the number of hospitalizations from 2016 to 2019. Numbers dipped in 2020, likely reflecting the impact of the COVID-19 pandemic on healthcare utilization and transmission dynamics. However, from 2021 onwards, rates recovered and started increasing again, peaking in the final year of study, 2023. Notably, similar post-pandemic increases have been reported in other pediatric infectious diseases, including pneumococcal disease, invasive group A streptococcus infection, and respiratory syncytial virus (RSV) infections, where a rebound was observed following the relaxation of public health measures (12–14). This phenomenon has been attributed to changes in population immunity, altered pathogen circulation, and increased susceptibility after a prolonged period of reduced exposure during the pandemic (12–14). Although these findings support a genuine increase in disease burden, the contribution of changes in diagnostic awareness, referral patterns, and healthcare utilization may also affect the observed magnitude of this trend.

The parallel increase observed in both hospitalization rates per admissions and per population suggests that the observed trend is unlikely to be explained solely by changes in hospital admission practices and may, at least in part, reflect a genuine increase in disease burden. Nevertheless, the administrative nature of the database does not allow direct evaluation of factors such as changes in diagnostic coding practices, clinical awareness, or healthcare utilization over time. Therefore, the observed increase is likely multifactorial and should be interpreted with cautio.

Comparisons with other European countries remain challenging because population-based epidemiological studies on SSSS are extremely limited. Most of the available European literature consists of case reports, outbreak descriptions, or single-center pediatric series rather than nationwide surveillance studies (1, 3, 4, 7, 15, 16). Nevertheless, reports from Greece have documented increasing numbers of SSSS cases associated with the emergence of toxin-producing *Staphylococcus aureus* clones, suggesting that changes in SSSS epidemiology may also be occurring elsewhere in Europe (17, 18). However, the absence of comparable nationwide datasets from other European countries precludes direct comparisons with our findings. Taken together, our results contribute valuable population-level evidence from Spain and help address an important gap in the European epidemiological literature on SSSS.

Although our study was not designed to investigate the microbiological mechanisms underlying the observed epidemiological trends, previous studies from other European countries have described localized increases in SSSS incidence associated with the emergence of specific *S. aureus* clones. These reports provide biological context for potential mechanisms underlying SSSS epidemiology but should not be interpreted as evidence that similar events occurred in Spain. Doudoulakakis et al. (17) reported a sharp rise in SSSS cases linked to the emergence of a toxinogenic, methicillin-susceptible *S. aureus* clone (ST121) carrying exfoliative toxin genes (eta/etb). In a related study from the same group, the emergence of a dominant community-associated ST121 clone, resistant to mupirocin and fusidic acid, was associated with an increasing number of skin and soft tissue infections, including SSSS, and characterized by a high prevalence of virulence factors such as exfoliative toxins and Panton–Valentine leukocidin (18). Together, these studies provide a plausible biological hypothesis for temporal changes in SSSS epidemiology.

However, our administrative database does not include microbiological or molecular typing data and therefore cannot determine whether similar clonal changes occurred in Spain during the study period. Therefore, our study should be interpreted as providing epidemiological evidence of changing hospitalization patterns rather than evidence supporting any specific biological mechanism. The potential contribution of emerging toxin-producing *S. aureus* clones remains hypothetical and cannot be inferred from our data. Future studies integrating nationwide epidemiological data with microbiological surveillance would be valuable to clarify the potential contribution of emerging toxinogenic *S. aureus* strains to temporal trends in SSSS hospitalizations. Such studies will be essential to determine whether the temporal trends observed in this study are associated with changes in circulating *S. aureus* strains or are primarily driven by other epidemiological or healthcare-related factors. Our finding that SSSS mainly affects young children is consistent with the existing body of evidence, which describes it as a predominantly pediatric disease, particularly affecting newborns and children under 5 years of age. This susceptibility has been attributed to immature immune responses, reduced renal clearance of circulating exfoliative toxins, and age-related differences in skin barrier biology. The predominance of cases in children aged 0–4 years observed in our study therefore aligns with the known pathophysiology of the disease (2, 6, 15, 16).

Although the relative increase in numbers was greater among children aged 5–14 years, the highest burden of disease was consistently concentrated in children aged 0–4 years across all study years. This finding reinforces the established epidemiology of SSSS and suggests that, despite temporal changes, the most vulnerable population has not changed its profile (16).

At the same time, our results highlight a clear age-related gradient in disease severity. While SSSS generally presented as a mild condition in children, with low mortality and short hospital stays—consistent with previous studies (1, 15, 16)—it was associated with significantly worse outcomes in adults, including higher rates of sepsis, organ failure, ICU admission, and mortality. These findings are in line with prior reports (5, 6, 19–21), which indicate that adult SSSS, although rare, is frequently associated with underlying comorbidities and carries a substantially higher risk of adverse outcomes. However, the relatively small number of adult cases included in our study (*n* = 84) should be taken into account when interpreting these findings, and larger studies are needed to confirm the observed differences between pediatric and adult populations.

In our cohort, this increased severity in adults was accompanied by a higher burden of comorbidities, including hypertension, chronic kidney disease, diabetes, neoplasia, and history of transplantation. Together, these findings support the conclusion that SSSS in adults often occurs in the context of systemic vulnerability and may represent a marker of severe underlying illness rather than an isolated dermatological condition.

Furthermore, within the pediatric population, younger age—particularly the first month of life—was associated with increased severity, as reflected by higher ICU admission rates, longer hospital stays, and the occurrence of mortality exclusively in children under one year of age (2, 15, 16, 22). Indeed, neonates represented the subgroup with the greatest disease severity in our study, showing the highest rates of ICU admission, the longest hospital stays, and the highest hospitalization costs. These findings highlight the particular vulnerability of newborns and reinforce the need for early recognition and prompt management of SSSS in this age group. This observation is biologically plausible and has been described elsewhere, as newborns are especially vulnerable due to immune immaturity, reduced renal clearance of toxins, and increased risk of nosocomial transmission (2, 16, 22). In neonatal settings, SSSS outbreaks have been linked to colonized healthcare workers or environmental reservoirs, highlighting the importance of strict infection control measures (2–4). However, in this population-based study, it was not possible to assess colonization status or transmission pathways, including the potential role of healthcare workers.

Regarding the economic burden, the total estimated cost of hospitalization over the study period was €4.42 million. Although the highest total cost was observed in younger children due to the greater number of hospitalizations, the median cost per admission was higher in adults (€4,087). This finding likely reflects the greater clinical complexity and severity of SSSS in adults, resulting in increased healthcare resource utilization, including longer hospital stays and higher rates of ICU admission.

Within the pediatric population, higher median costs were observed in newborns (€3,435), along with greater variability, indicating increased resource use in this subgroup, consistent with their higher rates of ICU admission and prolonged hospital stays. Overall, the cost of pediatric SSSS hospitalizations appears to be lower than that reported for other pediatric dermatological conditions, such as cellulitis in Spain (23), and markedly lower than the costs associated with adult cellulitis (24). This difference may be explained by the generally favorable clinical course of SSSS in children, which is typically associated with shorter hospital stays, low rates of complications, and limited need for invasive diagnostic or therapeutic procedures. In contrast, cellulitis frequently requires additional imaging studies, surgical interventions such as abscess drainage, and more heterogeneous management strategies, resulting in greater healthcare resource utilization and higher hospitalization costs. Importantly, cost estimates in both studies were derived using the same DRG-based methodology (23, 24), suggesting that the observed differences are unlikely to be attributable to variations in cost calculation methods.

A major strength of this study is its nationwide scope and the comprehensive age-stratified analysis, which provides a robust population-based overview of SSSS hospitalizations in Spain. The use of a national administrative database allowed the inclusion of a large sample size over an extended period, enabling reliable estimates of temporal trends, hospitalization rates, clinical characteristics, and outcomes across different age groups (23, 25).

However, several limitations should be acknowledged. First, as with all administrative databases, the unit of analysis is the hospital discharge, and each admission (including readmissions) is recorded as a separate episode, which may lead to a potential overestimation of cases (26). Second, the available clinical information is limited to coded discharge diagnoses, and variability in coding practices across centers may entail misclassifications or inconsistencies. Third, microbiological data are based on diagnostic coding and do not include detailed information on culture results, toxin identification, or sample origin, which limits pathogen-specific interpretation. Furthermore, the database does not contain microbiological culture results, antimicrobial susceptibility patterns, or molecular typing data. Consequently, we could not evaluate the role of specific *Staphylococcus aureus* clones or toxin profiles in the epidemiological patterns observed. Fourth, the database does not include laboratory parameters (e.g., inflammatory markers, kidney function) or detailed clinical variables, preventing a more granular assessment of disease severity.

Fifth, although this nationwide study included a large overall sample of hospitalized patients with SSSS, only 84 adult cases were identified during the study period. Consequently, comparisons involving the adult subgroup should be interpreted with caution, as the limited sample size reduces the statistical precision of estimates and the generalizability of findings in adults. Furthermore, this study only captures hospitalized patients and therefore reflects the more severe end of the SSSS disease spectrum. Mild cases managed in outpatient settings, primary care, or emergency departments without hospital admission are not included in the database. Consequently, the findings should not be interpreted as representative of the total burden of SSSS in the general population. Finally, information on therapeutic management, including antibiotic regimens, supportive care, or infection control measures, is not available, which precludes analysis of treatment strategies and their impact on outcomes.

This study highlights an increasing trend in SSSS hospitalizations in Spain, with a temporary decline during the COVID-19 pandemic followed by a marked rise thereafter. The highest burden of disease was consistently observed in young children, particularly those aged 0–4 years. Within the pediatric population, most cases had a favorable clinical course, with low mortality and limited need for ICU admission. However, younger children—especially newborns and infants—showed greater severity, including higher ICU admission rates, longer hospital stays, and the only deaths observed in the pediatric group.

In contrast, although less frequently affected, adults presented with a higher burden of comorbidities and significantly worse outcomes, emphasizing that SSSS represents a severe and potentially life-threatening condition in this population. Overall, these findings underscore the importance of continued epidemiological surveillance and highlight the value of nationwide administrative databases for monitoring trends and outcomes in rare infectious diseases.

## Data Availability

The raw data supporting the conclusions of this article will be made available by the authors, without undue reservation.
